# Influence of Metal Composition and Support Material on Carbon Yield and Quality in the Direct Decomposition of Methane

**DOI:** 10.3390/molecules30091903

**Published:** 2025-04-24

**Authors:** Uidam Jun, Bon-Jun Ku, Yeji Gwon, Dong-Hyun Kim, Mansu Kim, I-Jeong Jeon, Hongjin Lee, Jae-Oh Shim, Kyubock Lee

**Affiliations:** 1 Graduate School of Energy Science and Technology, Chungnam National University, 99 Daehak-ro, Yuseong-gu, Daejeon 34134, Republic of Korea; doe06019@gmail.com (U.J.); kubj9333@gmail.com (B.-J.K.); izjey408@gmail.com (Y.G.); donghyun7372@gmail.com (D.-H.K.); leehj@kier.re.kr (H.L.); 2 Department of Chemistry, Northwestern University, Evanston, IL 60208, USA; mansu.kim@northwestern.edu; 3 Department of Chemical Engineering, Wonkwang University, 460 Iksan-daero, Iksan-si 54538, Republic of Korea; dlwjd7448@wku.ac.kr (I.-J.J.); joshim85@wku.ac.kr (J.-O.S.); 4 Hydrogen Department, Korea Institute of Energy Research, 152 Gajeong-ro, Yuseong-gu, Daejeon 34129, Republic of Korea

**Keywords:** direct decomposition of methane, spray pyrolysis, carbon nanotube, Ni-Cu/MgO, Ni/Al_2_O_3_

## Abstract

A series of catalysts were synthesized via a combination of evaporation-induced self-assembly and spray pyrolysis; they were then applied to the direct decomposition of methane. Among them, Ni-Cu/MgO catalysts exhibited the smallest Ni particle size (~9 nm), attributed to the Cu-induced suppression of Ni crystal growth during synthesis. These catalysts achieved the highest carbon yield, primarily due to the enhanced dispersion and nanoscale size of Ni particles. The interaction between methane and the catalysts, as well as the structural and electrical properties of the resulting carbon nanotubes, such as crystallinity and conductivity, were investigated with respect to the support material (MgO vs. Al_2_O_3_) and metal composition (Ni vs. Ni-Cu). The findings provide valuable insights for designing advanced catalyst systems for the efficient conversion of methane into high-value carbon-based materials.

## 1. Introduction

The direct decomposition of methane (DDM) is a catalytic process in which methane (CH_4_) is directly converted into hydrogen (H_2_) and solid carbon (C) without producing carbon oxides (CO_x_). A major advantage of this method lies in its minimal greenhouse gas emissions. In contrast, conventional hydrogen production routes such as steam methane reforming and partial oxidation emit significant amounts of CO_2_—typically, at least 1 mole per mole of methane consumed [1,2,3,4]. Beyond hydrogen, DDM also enables the co-production of valuable carbon nanomaterials such as carbon nanotubes (CNT) with broad applications in gas storage [5], polymer reinforcement [6], and catalysis or catalyst supports [7]. However, due to the high symmetry and strong C-H bonds in methane (bond energy ~440 kJ/mol), thermal decomposition typically requires temperatures exceeding 1200 °C [8]. To lower the activation energy and enhance methane conversion, metal-based catalysts have been extensively investigated [1,9,10].

A wide range of mono- and bimetallic catalysts—including Ni, Cu, Co, Mo, Fe and their combinations (e.g., Ni-Cu, Co-Mo, Fe-Ni)—have been studied for their DDM performance [11,12,13,14,15,16,17,18,19,20,21,22]. For instance, Ni/Al_2_O_3_ catalysts with high nickel loading (40 wt.%) have achieved carbon yields as high as 15 g C/g catalyst at 600 °C due to the increased availability of active metal sites [11]. The incorporation of Cu (e.g., 5 wt.% in 50 wt.% Ni catalysts) has been shown to enhance Ni reducibility and promote carbon yields (9 g C/g at 650 °C) [18]. Similarly, adding Mo to Co/MgO catalysts has led to remarkable carbon yields (~40 g C/g), facilitated by MgMoO_4_ phases that improve cobalt dispersion and stabilize active sites while promoting the aromatization of hydrocarbons for CNT formation [19]. Catalyst supports also play a critical role in modulating catalyst performance. Supports such as Al_2_O_3_, SiO_2_, and MgO influence the electronic and structural properties of active metals through metal–support interactions [23]. For example, strong interactions between Co and Al_2_O_3_ can lead to the formation of CoAl_2_O_4_ spinel phases, which help maintain high dispersion and suppress sintering [24]. Moreover, support morphology and surface chemistry significantly affect the characteristics of carbon products, enabling the selective synthesis of structures such as vertically aligned CNT [17,25,26,27].

While many studies have focused on optimizing individual catalyst components or synthesis conditions, systematic comparisons across different compositions synthesized using a unified method are scarce. In this study, a set of catalyst formulations previously reported to exhibit high performance in DDM were synthesized using a consistent methodology—combining evaporation-induced self-assembly (EISA) with spray pyrolysis—to ensure high metal dispersion and uniform metal–support interactions. The catalytic activities of catalysts were evaluated under the optimized condition for each catalyst. Temperature-programmed surface reactions and desorption studies were conducted for a better understanding of methane activation behavior and the available active sites of the catalysts, respectively. Furthermore, the quality of the carbon products (mainly focusing on crystallinity and electron conductivity) grown from the catalytic reaction was investigated using Raman analysis and electrical conductivity measurements, revealing the influence of factors such as the metal particle size, support type (Al_2_O_3_ vs. MgO), and copper loading of the catalysts.

## 2. Results and Discussion

### 2.1. Catalyst Screening Based on Carbon Yields

The chemical compositions of the catalysts used in this study are listed in Table 1. These compositions were selected based on previous reports of high carbon yield performance in DDM [11,13,17,18,19,21]. Six candidate catalysts were synthesized via a one-pot spray pyrolysis method and initially screened under DDM conditions. Among them, Ni/Al_2_O_3_ and Ni–Cu/MgO exhibited the highest carbon weight gain and were therefore selected for further in-depth analysis.

### 2.2. Catalyst Physicochemical Properties

**Figure 1:**
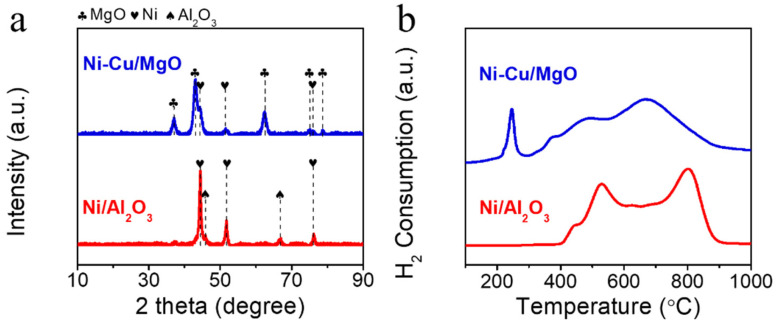
(**a**) XRD patterns of reduced catalysts and (**b**) H_2_-TPR profiles of calcined catalysts.

The crystalline structures of the reduced catalysts were analyzed using X-ray diffraction (XRD), as shown in Figure 1a. Diffraction peaks corresponding to Al_2_O_3_ (JCPDS 29-0063), MgO (JCPDS 45-0946), and metallic Ni (JCPDS 04-0850) were identified [28,29,30]. Both Ni/Al_2_O_3_ and Ni-Cu/MgO exhibited distinct Ni^0^ peaks, consistent with their high Ni content. While high metal loading can promote nanoparticle aggregation, the spray pyrolysis method effectively suppressed excessive sintering, leading to well-dispersed particles. The average Ni crystallite sizes, estimated via the Scherrer equation using the Ni^0^ (100) reflection at 44.5°, were 15.6 nm for Ni/Al_2_O_3_ and 9.0 nm for Ni-Cu/MgO (Table 2). According to previous reports using the same chemical compositions as in our catalyst preparation, the Ni particle size was 24 nm for the Ni/Al_2_O_3_ catalyst and 59 nm for the Ni-Cu/MgO catalyst [11,18]. The smaller particle size in the Cu-doped catalyst is attributed to the Cu-induced disruption of Ni diffusion during synthesis, suppressing growth and enhancing dispersion [31,32].

H_2_-temperature-programmed reduction (H_2_-TPR) profiles (Figure 1b) revealed the reducibility characteristics of the catalysts. Ni/Al_2_O_3_ exhibited two major reduction regions: bulk NiO reduction (350–650 °C) and Ni-aluminate reduction (>650 °C), indicating strong metal–support interactions [33]. In contrast, Ni-Cu/MgO showed multiple peaks at 245, 482, and 671 °C. While Ni reduction on MgO typically occurs above 650 °C [34], the addition of Cu induced a shift to lower temperature (482 °C) via the hydrogen spillover effect, which enhances H_2_ dissociation and accelerates Ni reduction [18]. This promoted reducibility correlates with the smaller Ni crystallites observed via XRD.

**Figure 2:**
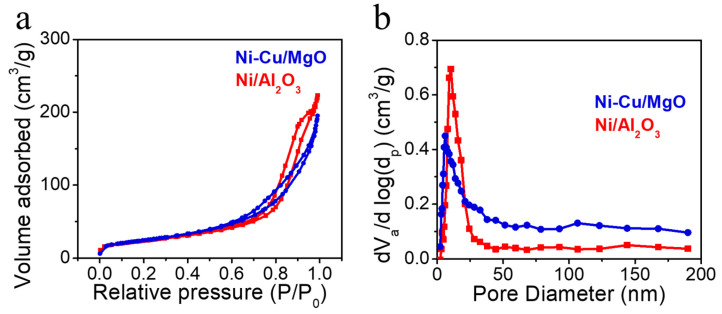
(**a**) Nitrogen adsorption–desorption isotherms, and (**b**) BJH pore size distributions of the reduced catalysts.

The porosity and surface area of the catalysts were evaluated using N_2_ adsorption-desorption (Figure 2). Ni/Al_2_O_3_ displayed an H5-type isotherm, characteristic of blocked mesopores, whereas Ni-Cu/MgO exhibited an H3-type isotherm, suggesting slit-shaped pores or plate-like structures [35]. Both catalysts showed mesoporosity, with pore sizes mainly between 5–20 nm and surface areas ranging from 80–90 m^2^/g after reduction (Table 3), indicating that the porous structures were preserved post-calcination.

**Figure 3:**
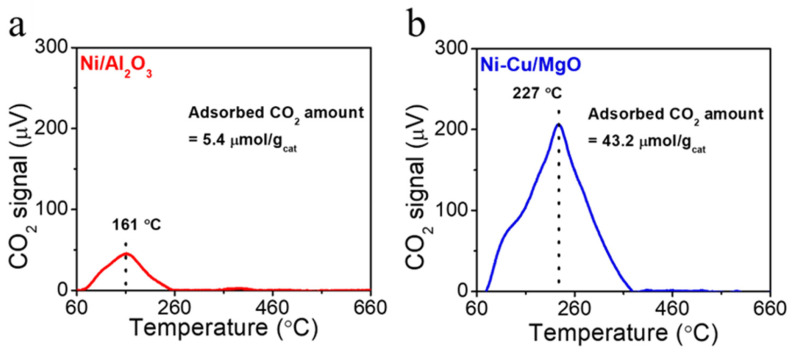
CO_2_-TPD profiles of (**a**) Ni/Al_2_O_3_ and (**b**) Ni-Cu/MgO catalysts.

The surface basicity of the pre-reduced catalysts was investigated using the temperature-programmed desorption of CO_2_ (CO_2_-TPD), and the results are shown in Figure 3. The desorption profile of the Ni/Al_2_O_3_ catalyst (Figure 3a) exhibited a weak low-temperature peak centered at 161 °C, which can be attributed to physiosorbed CO_2_ or weak basic sites associated with surface hydroxyl groups on Al_2_O_3_ [36]. In contrast, the Ni-Cu/MgO catalyst showed a desorption peak centered at 227 °C, corresponding to moderate basic sites [37]. This peak is assigned to chemisorbed carbonate species formed via the interaction of CO_2_ with surface O^2−^ anions on MgO. The total amount of CO_2_ adsorbed was significantly higher for Ni-Cu/MgO (43.2 μmol/g_cat_) than for Ni/Al_2_O_3_ (5.2 μmol/g_cat_), indicating a much higher density of basic sites. The strong basicity of the support MgO can enhance the dispersion and stabilization of metal nanoparticles [38].

### 2.3. Catalytic Activity in DDM

**Figure 4:**
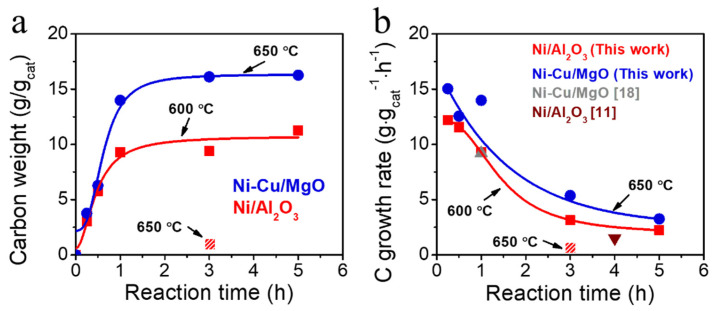
Carbon production over catalysts. (**a**) Carbon weight gain as a function of reaction time and (**b**) hourly carbon weight ratio as a function of reaction time. DDM reaction conditions: 50% CH_4_/N_2_, GHSV = 120 L·g_cat_·h^−1^, catalyst loading = 0.2 g.

The carbon growth performance under DDM conditions is shown in Figure 4. The DDM experiments were conducted for various reaction times under the optimum temperature conditions for each catalyst (650 °C for Ni-Cu/MgO and 600 °C for Ni/Al_2_O_3_), as determined in previous studies. Additionally, to enable a comparison with Ni-Cu/MgO under identical temperature conditions, the Ni/Al_2_O_3_ catalyst was tested at 650 °C for three hours. Since this temperature is not optimal for Ni/Al_2_O_3_, a significantly lower carbon yield was observed. When both Ni/Al_2_O_3_ and Ni-Cu/MgO were operated at their respective optimum temperatures, rapid carbon accumulation occurred in the early stages of the reaction (Figure 4a), with yields approaching near saturation within one hour. The final carbon yields were ~9 g/g_cat_ for Ni/Al_2_O_3_ and ~16 g/g_cat_ for Ni-Cu/MgO. Compared to previously reported values for similar compositions [11,18], the catalysts prepared in this study achieved higher carbon yields (Figure 4b), likely due to enhanced metal dispersion and the reduced Ni particle size enabled by the spray pyrolysis synthesis.

**Figure 5:**
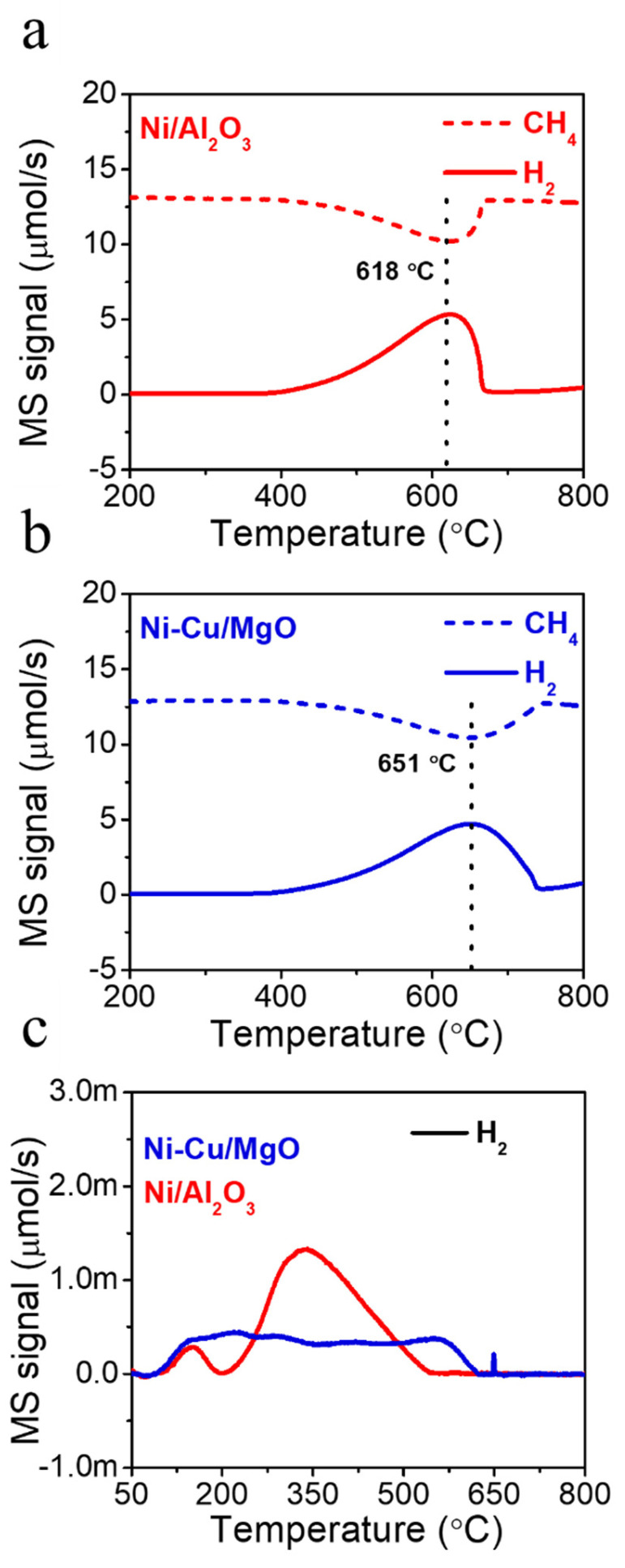
CH_4_-TPSR MS profiles of (**a**) Ni/Al_2_O_3_ and (**b**) Ni-Cu/MgO catalysts. CH_4_-TPSR condition = 50% CH_4_/N_2_, GHSV = 240 L·g_cat_·h^−1^, catalyst loading = 0.01 g. (**c**) CH_4_-TPD MS profiles of two catalysts.

To examine CH_4_ activation behavior, CH_4_-temperature-programmed surface reaction (CH_4_-TPSR) experiments were performed (Figure 5a,b). CH_4_ conversion and H_2_ evolution occurred at 618 °C (Ni/Al_2_O_3_) and 651 °C (Ni-Cu/MgO). The higher activation temperature observed for Ni-Cu/MgO may result from Cu’s intrinsic inactivity toward CH_4_ decomposition, which shifts the CH_4_ dissociation to a higher temperature range. This trend aligns with previous reports produced by Wang et al. [22], who observed that the addition of Cu increases the CH_4_ decomposition temperature but enhances carbon yields via improved dispersion and alloy formation. In other studies, the incorporation of copper into Ni-based catalysts changed the catalyst particle morphology from cuboctahedral to quasi-octahedral, promoting filamentous carbon growth and thereby enhancing the carbon yield [14].

**Figure 6:**
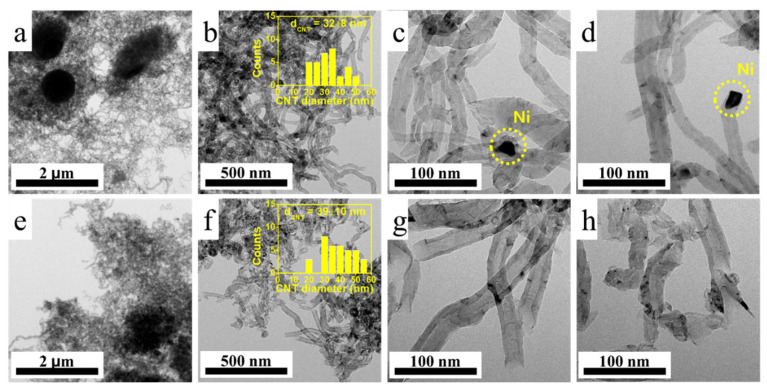
TEM images and CNT diameter distribution for the used catalysts. (**a**–**d**) TEM images of Ni/Al_2_O_3_ and (**e**–**h**) Ni-Cu/MgO catalysts. The CNT diameter distribution was determined based on 30 measured counts.

CH_4_-temperature-programmed desorption (CH_4_-TPD) analysis (Figure 5c) was used to examine active site availability. Only H_2_ was detected, indicating the full dissociation of CH_4_ without the desorption of intact molecules. The CH_4_ uptake, inferred from the H_2_ evolution, was 12.5 nmol/g_cat_ for Ni/Al_2_O_3_ and 8.7 nmol/g_cat_ for Ni-Cu/MgO. Despite the lower CH_4_ uptake, Ni-Cu/MgO achieved a higher carbon yield, which can be attributed to more efficient carbon precipitation pathways [22]. However, the actual carbon yield is influenced not only by CH_4_ adsorption but also the surface properties of active sites that can dissociate CH_4_ and facilitate carbon precipitation. The H_2_ evolution in Ni/Al_2_O_3_ was initiated at lower temperatures (300–500 °C), while Ni-Cu/MgO exhibited broader H_2_ release over a range. This suggests differing C–H bond activation mechanisms, potentially due to the bimetallic nature of Ni-Cu/MgO [39]. Furthermore, basic sites—particularly O^2^⁻ species on MgO supports from Ni-Cu/MgO—can interact with hydrogen atoms from CH_4_, thereby promoting C-H bond cleavage. This interaction facilitates CH_4_ activation over a broader or even lower temperature range, especially when combined with active metal sites such as Ni [40]. These results highlight the critical role of particle dispersion and composition in catalytic performance. The morphology of carbon deposits was analyzed using TEM (Figure 6). Metal nanoparticles were scarcely observed on the tip of CNT in both catalysts. Specifically, the Ni-Cu/MgO catalyst did not exhibit any noticeable Ni particles at the CNT tips, and the Ni/Al_2_O_3_ catalyst showed only very rare occurrences of metal particles. This implies a base-growth mechanism in which CNT grows upwards while the catalyst remains anchored on the support [41]. This mode of growth provides catalyst stability and enables continuous CNT formation.

### 2.4. Characterization of CNT

The average diameters of CNT, measured from TEM images (Figure 6b,f), ranged from 30 to 40 nm for both Ni/Al_2_O_3_- and Ni-Cu/MgO-derived samples. Longer CNTs were observed in the Ni/Al_2_O_3_-derived samples. As discussed earlier, the Ni-Cu/MgO catalyst exhibited a smaller Ni grain size than that of the Ni/Al_2_O_3_ catalyst. However, the CNTs synthesized using Ni/Al_2_O_3_ exhibited smaller diameters. Although many previous studies have suggested a correlation between catalyst particle size and CNT diameter [42,43], such a correlation was not observed in the present study. Moodley et al. reported that the relationship between particle size and CNT diameter is not always consistent during CNT synthesis, as particle rearrangements such as coalescence and redispersion can occur under reaction conditions [44,45].

**Figure 7:**
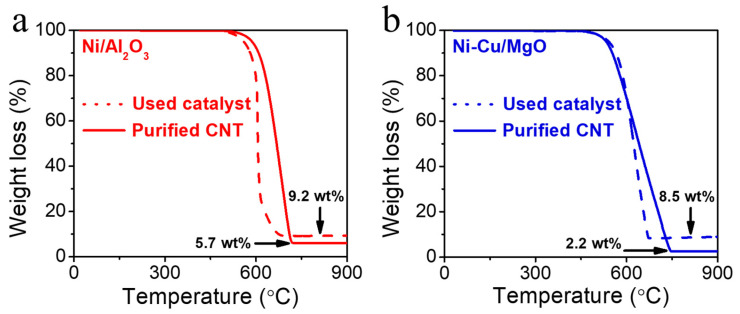
TGA analysis of the used catalysts and purified CNTs after acid treatment: (**a**) Ni/Al_2_O_3_ and (**b**) Ni-Cu/MgO.

To evaluate the properties and purity of CNTs, catalysts after reactions were acid-treated and analyzed via thermogravimetric analysis (TGA) (Figure 7). For Ni-Cu/MgO, only less than 2.2 wt% of residue was left after full oxidation, indicating effective impurity removal and high CNT purity. This is attributed to the use of MgO as a support, which can be easily removed through a mild acid treatment without damaging the carbon structure. In contrast, Al_2_O_3_ is more chemically inert and difficult to dissolve, even in strong acids. The removal of Al_2_O_3_ often requires harsh conditions that can negatively affect the CNTs. Therefore, MgO is often preferred as a support in carbon synthesis [46].

**Figure 8:**
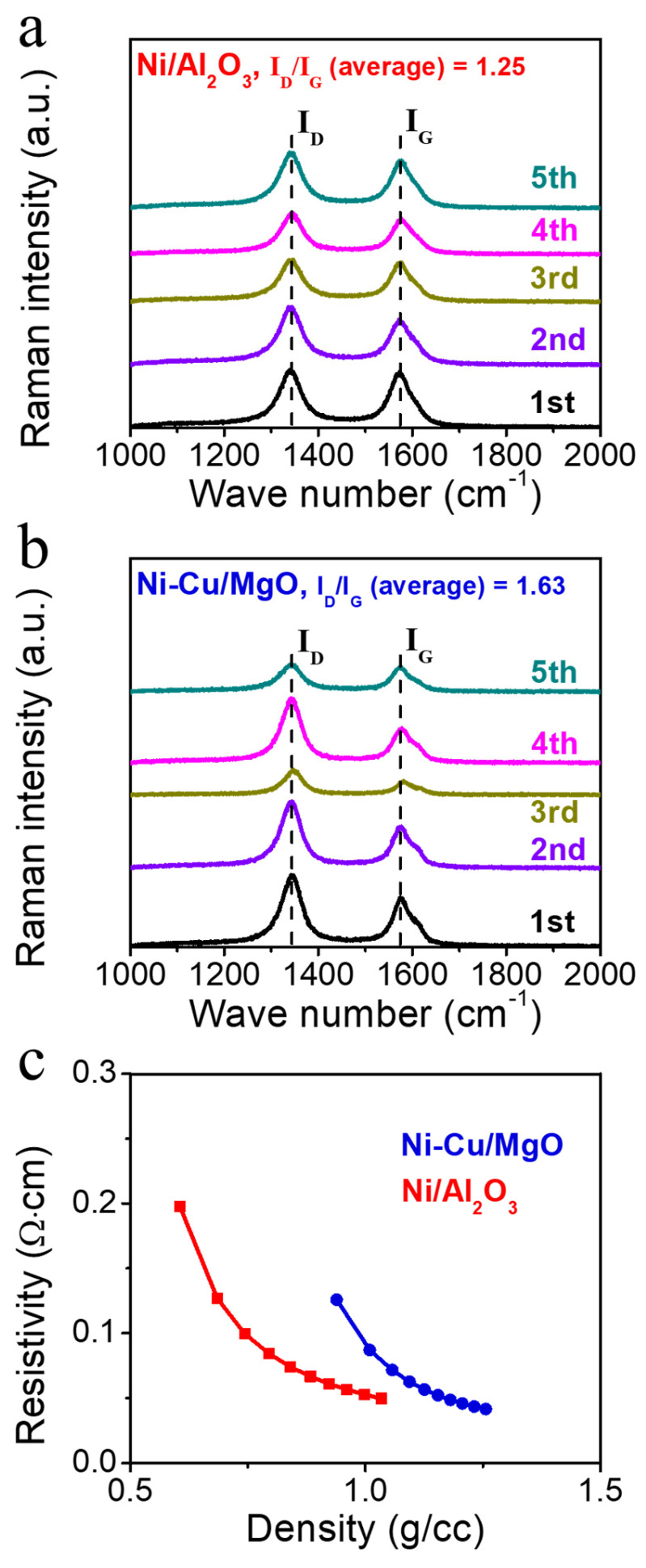
Raman spectra of purified CNT. (**a**) Raman spectra of CNT grown from Ni/Al_2_O_3_ and (**b**) Ni-Cu/MgO catalysts. (**c**) Powder resistivity analysis of CNT.

Raman spectra of purified CNTs (Figure 8a,b) displayed prominent D and G bands at 1340 and 1583 cm^−^^1^, respectively. The intensity ratio (I_D_/I_G_), reflecting the degree of graphitization [47], was 1.25 for the Ni/Al_2_O_3_-derived CNTs and 1.63 for those from Ni-Cu/MgO, suggesting higher crystallinity in the former. CNTs with higher crystallinity typically exhibit enhanced electrical conductivity, mechanical strength, and thermal stability, making them more suitable for application [10]. The existence of less ordered CNTs may be due to uneven carbon diffusion on the Ni-Cu surface [48]. The varying carbon solubility in the Cu segregation areas has been reported to promote non-uniform graphitic growth and lower crystallinity.

The electrical conductivity of CNTs is important due to its direct impact on their electrical performance in various applications, including electronics, sensors, energy storage devices, and conductive composites. Electrical resistivity measurements (Figure 8c) showed that CNTs from Ni/Al_2_O_3_ exhibited lower resistivity than those from Ni-Cu/MgO. This is attributed to their higher crystallinity, which facilitates charge transport by minimizing defects and forming continuous, well-connected conductive networks [49,50,51]. The longer CNTs observed in Ni/Al_2_O_3_ samples also contribute to improved conductivity by reducing inter-tube resistance. These findings suggest that the choice of catalyst and support strongly influences the physical and electrical characteristics of CNTs. Catalysts that promote the formation of long, well-graphitized CNTs are therefore more favorable for applications requiring high electrical conductivity (Table 4).

## 3. Materials and Methods

### 3.1. Materials

The following metal precursors were used as received, without further purification: nickel nitrate hexahydrate (Ni(NO_3_)_2_·6H_2_O, ≥98%, Samchun, Republic of Korea), aluminum nitrate nonahydrate (Al(NO_3_)_3_·9H_2_O, ≥98%, Samchun, Republic of Korea), copper nitrate trihydrate (Cu(NO_3_)_2_·3H_2_O, ≥98%, Sigma-Aldrich, St. Louis, MO, USA), magnesium nitrate hexahydrate (Mg(NO_3_)_2_·6H_2_O, ≥98%, Sigma-Aldrich, St. Louis, MO, USA), cobalt nitrate hexahydrate (Co(NO_3_)_2_·6H_2_O, Extra pure, Samchun, Republic of Korea), iron nitrate nonahydrate (Fe(NO_3_)_3_·9H_2_O, Extra pure, Samchun, Republic of Korea), and ammonium molybdate ((NH_4_)_6_Mo_7_O_24_·4H_2_O, ≥99.98%, Sigma-Aldrich, St. Louis, MO, USA). Tetraethyl orthosilicate (TEOS, (C_2_H_5_O)_4_Si, >97.0%, Sigma-Aldrich, St. Louis, MO, USA) was used as a silica source. Pluronic P123 (EO_20_PO_70_EO_20_, MW = 5800 g/mol, Sigma-Aldrich, St. Louis, MO, USA) served as a structure-directing agent. Nitric acid (HNO_3_, ≥60%, Samchun, Republic of Korea) and anhydrous ethanol (C_2_H_5_OH, ≥99.9%, Samchun, Republic of Korea) were used as solvents.

### 3.2. Catalyst Preparation

Catalysts including Ni/SiO_2_ [21], Co-Mo/MgO [19], Ni-Cu/MgO [18], Fe/Al_2_O_3_ [17], Ni/Al_2_O_3_ [11], and Ni/MgO [13] were synthesized using a spray pyrolysis-assisted one-pot EISA method. First, 15 g of P123 was dissolved in a solution containing 460 mL of deionized water, 500 mL of ethanol, and 40 g of HNO_3_. Stoichiometric amounts of metal precursors were then added to this solution as follows:

Ni/SiO_2_: 2.477 g of Ni(NO_3_)_2_·6H_2_O, 31.208 g of TEOS

Co-Mo/MgO: 1.315 g of Co(NO_3_)_2_·6H_2_O, 3.190 g of (NH_4_)_6_Mo_7_O_24_·4H_2_O, 50.894 g of Mg(NO_3_)_2_·6H_2_O

Ni-Cu/MgO: 24.772 g of Ni(NO_3_)_2_·6H_2_O, 1.901 g of Cu(NO_3_)_2_·3H_2_O, 28.628 g of Mg(NO_3_)_2_·6H_2_O

Fe/Al_2_O_3_: 20.980 g of Fe(NO_3_)_3_·9H_2_O, 52.244 g of Al(NO_3_)_3_·9H_2_O

Ni-Al_2_O_3_: 19.818 g of Ni(NO_3_)_2_·6H_2_O, 44.150 g of Al(NO_3_)_3_·9H_2_O

Ni-MgO: 36.036 g of Ni(NO_3_)_2_·6H_2_O, 30.893 g of Mg(NO_3_)_2_·6H_2_O

The precursor solution was aerosolized using a 1.7 MHz ultrasonic nebulizer equipped with six piezoelectric vibrators, and the mist was carried into a quartz tube reactor maintained at 700 °C by a 10 L·min^−1^ flow of N_2_ gas. The resulting particles were collected via a bag filter heated at 100–130 °C. Calcination was carried out in air conditions at the following temperatures for each catalyst as indicated in each reference: 400 °C for Ni/SiO_2_, 800 °C for Co-Mo/MgO, 500 °C for Ni-Cu/MgO, 750 °C for Fe/Al_2_O_3_, 800 °C for Ni- Al_2_O_3_, 500 °C for Ni-MgO.

### 3.3. Catalytic Reaction

Catalytic DDM was conducted in a horizontal tube furnace using a three-inch internal diameter quartz tube. Catalysts were uniformly spread in ceramic boats and placed at the center of the tube. Prior to the reaction, the system was purged with 200 mL·min^−1^ of N_2_ for 15 min. Each catalyst was subjected to reduction and reaction treatments at the respective temperatures reported in the corresponding references, as summarized below: Ni/SiO_2_ (reduction at 400 °C/reaction at 550 °C), Co-Mo/MgO (800 °C/700 °C), Ni-Cu/MgO (550 °C/650 °C), Fe/Al_2_O_3_ (700 °C/700 °C), Ni-Al_2_O_3_ (800 °C/600 °C), and Ni-MgO (500 °C/670 °C).

The reduction step was performed for 2 h under 4% H_2_ in Ar at a flow rate of 400 mL·min^−1^. After reduction, the reactor was purged with 400 mL·min^−1^ of N_2_ for 1 h during ramping to the reaction temperature. During the reaction, CH_4_ and N_2_ were introduced at 200 mL·min^−1^ each. Reactions were conducted for durations ranging from 0.25 to 5 h.

### 3.4. Acid Treatment for the Purification of CNT

After the reaction, catalysts reacted for 3 h were ground to fine powders. For the acid treatment, 100 mg of the sample was treated in 50 mL of 1 M HCl at 70 °C with vigorous stirring for 5 h. The resulting suspension was washed via repeated centrifugation with DI water until the pH reached 7.0. The purified CNTs were dried in oven at 80 °C overnight and then collected.

### 3.5. Catalyst Characterization

Catalyst characterization was carried out using a combination of physicochemical and spectroscopic techniques. The specific surface area and pore size distribution were analyzed using the Brunauer–Emmett–Teller (BET) and the Barrett–Joymer–Halenda BJH methods, respectively, using a BELSORP-mini (MicrotracBEL, Osaka, Japan). In brief, 50 mg of each sample was introduced in the dedicated cell and analyzed after degassing at 300 °C for 2 h under vacuum. Crystalline phases were identified by X-ray diffraction (XRD) using a SmartLab 9 kW diffractometer (RIGAKU, Tokyo, Japan) with Cu Kα radiation, scanning the 2θ range from 10° to 90° at a rate of 1°/min. H_2_-TPR, CH_4_-TPD, CO_2_-TPD, and CH_4_-TPSR were performed using a BELCAT II (MicrotracBEL, Japan). Then, 100 mg of each sample was located between 100 mg of quartz wool in the dedicated cell. The prepared cell was inserted into the equipment, and this was followed by pretreatment and analysis. Hydrogen-temperature-programmed reduction (H_2_-TPR) was conducted over a temperature range of 50–1000 °C under 20% H_2_ in Ar (10 °C·min^−1^), following a pre-treatment at 250 °C for 2 h. To estimate the number of active sites, CH_4_-temperature programmed desorption (CH_4_-TPD) was performed after CH_4_ adsorption at 50 °C for 30 min, followed by heating to 800 °C under Ar at 5 °C·min^−1^. CO_2_-temperature programmed desorption (CO_2_-TPD) was carried out after CO_2_ adsorption at 50 °C for 60 min, then heated to 1000 °C under an argon flow at a rate of 10 °C·min^−1^. A methane-temperature-programmed surface reaction (CH_4_-TPSR) was carried out on 10 mg of catalyst loaded with quartz wool, with a heating ramp of 5 °C·min^−1^ up to 800 °C under a flow of 20 sccm CH_4_ and 20 sccm Ar, following pre-treatment at 250 °C under Ar condition. Thermogravimetric analysis (TGA) in air was employed to quantify the carbon content in both the spent and acid-purified catalysts. Raman spectroscopy of the purified CNTs was performed using a LabRam Soleil system (Horiba, Longjumeau, France), and their electrical resistivity was measured using an HPRM-FA2 unit (HANTECH, Gimhae-Si, Republic of Korea). Morphological observations of the catalysts and CNTs were conducted via transmission electron microscopy (TEM) using a Tecnai G2 F30 instrument (FEI, Hillsboro, OR, USA).

## 4. Conclusions

This study evaluated the productivity and the quality of CNTs grown from a series of catalysts formulated based on previously reported compositions known for high carbon production in the DDM reaction. All catalysts were synthesized using a unified approach that combined EISA with spray pyrolysis, allowing for systematic comparisons across different formulations. The enhanced carbon yield observed for the Ni-Cu/MgO catalyst was primarily attributed to the formation of smaller Ni nanoparticles, enabled by the addition of Cu, which disrupted Ni crystallization and growth during synthesis. Furthermore, the basic nature of the MgO support contributed to improved metal dispersion, even at high Ni loading (45 wt%). However, the characterization of the resulting CNTs revealed that those produced from Ni-Cu/MgO exhibited lower crystallinity and electrical conductivity compared to CNTs derived from the Ni/Al_2_O_3_ catalyst. These findings underscore the importance of balancing carbon yields and carbon quality, offering valuable guidance for the design of supported metal catalysts for methane conversion to value-added carbon materials.

## Figures and Tables

**Table 1:** Chemical composition of catalysts and their carbon yields from the DDM reaction.

Catalysts	Contents (wt%)	Reaction Time (h)	Carbon Yield (g·g_cat_^−1^·h^−1^)
Ni/Al_2_O_3_	Ni (40), Al_2_O_3_ (60)	3	3.13
Ni-Cu/MgO	Ni (50), Cu (5), MgO (45)	3	5.37
Co-Mo/MgO	Co (3), Mo (17), MgO (80)	3	0.31
Ni/SiO_2_	Ni (10), SiO_2_ (90)	3	0
Fe/Al_2_O_3_	Fe (29), Al_2_O_3_ (71)	3	0.26
Ni/MgO	Ni (71), MgO (29)	3	0.12

**Table 2:** Metal crystallite size of reduced catalysts.

Catalysts	Crystallite Size (nm)
Ni/Al_2_O_3_	15.64
Ni-Cu/MgO	9.04

**Table 3:** BET specific surface area (S.S.A.), total pore volume (T.P.V.) and BJH mean pore diameter (M.P.D).

Catalysts	S.S.A. (m^2^ g_cat_^−1^)	T.P.V. (cm^3^ g_cat_^−1^)	M.P.D. (nm)
Ni/Al_2_O_3_	85.53	0.34	16.01
Ni-Cu/MgO	87.03	0.30	13.86

**Table 4:** Comparison of key performance metrics of catalysts.

Catalysts	CNT Yield at 1 h Reaction (g·g_cat_^−1^) ^a^	CNT Diameter (nm) ^b^	CNT Crystallinity (I_D_/I_G_) ^c^	CNT Resistivity at 1 g/cc Density (Ω·cm) ^d^
Ni/Al_2_O_3_	9.29	32.36	1.25	0.05
Ni-Cu/MgO	13.99	38.61	1.63	0.09

^a^ Measured via DDM reaction. ^b^ Calculated by counting 40 CNTs from TEM micrographs. ^c^ Determined using Raman analysis. ^d^ Determined using a resistivity measurement system.

## Data Availability

The original contributions presented in this study are included in the article. Further inquiries can be directed to the corresponding author(s).
